# A130 EVALUATING PLACEBO RATES IN EOSINOPHILIC ESOPHAGITIS RANDOMIZED CONTROLLED TRIALS: A SYSTEMATIC REVIEW AND META-ANALYSIS

**DOI:** 10.1093/jcag/gwae059.130

**Published:** 2025-02-10

**Authors:** N S Ahmed, A Rivas, Y Yuan, A Qasim, D O’Gorman, B Feagan, V Jairath, A Bredenoord, E S Dellon, C Ma

**Affiliations:** University of Calgary Cumming School of Medicine, Calgary, AB, Canada; University of Calgary Cumming School of Medicine, Calgary, AB, Canada; London Health Sciences Centre, London, ON, Canada; Alimentiv Inc, London, ON, Canada; Alimentiv Inc, London, ON, Canada; London Health Sciences Centre, London, ON, Canada; Alimentiv Inc, London, ON, Canada; Amsterdam UMC Locatie AMC, Amsterdam, Noord-Holland, Netherlands; University of North Carolina School of Medicine, Chapel Hill, NC; Alimentiv Inc, London, ON, Canada

## Abstract

**Background:**

Eosinophilic esophagitis (EoE) is a chronic inflammatory and progressive fibrotic condition of the esophagus. There are limited treatment options for EoE and a substantial unmet need for effective and safe medical therapies. Drug development in EoE has been hampered by heterogeneous placebo response rates and uncertainty regarding appropriate endpoint configurations. Accurate characterization of placebo response in EoE randomized controlled trials (RCTs) will help inform more future efficient trial designs.

**Aims:**

To conduct a systematic review and meta-analysis to characterize symptomatic, endoscopic, and histologic placebo response rates in placebo controlled EoE RCTs.

**Methods:**

We updated a Cochrane systematic review and meta-analysis, searching MEDLINE, EMBASE, and CENTRAL to January 1, 2024, to identify placebo-controlled RCTs evaluating medical therapies for patients with EoE. Studies were screened independently by 2 reviewers, and full texts were then reviewed for eligibility; all data extracted in parallel. Primary outcome: pooled proportion of clinical, endoscopic, and histologic responders and remitters randomized to placebo, using an intention-to-treat approach and random-effects model. Potential sources of heterogeneity were explored using meta-regression.

**Results:**

A total of 25 RCTs were included. The pooled proportion of clinical response was 40.9% [95% CI: 29.7%-52.8%] with substantial heterogeneity (*I*^*2*^=74.9%). On meta-regression, older age and a higher probability of being randomized to placebo reduced the likelihood of clinical response to placebo. The pooled proportion of histologic remission defined as a peak eosinophil count [PEC] ≤6 eosinophils per high power field [HPF] or ≤1 eos/HPF was 4.3% [95% CI: 2.6%-6.2%] (*I*^*2*^=23.6%) and 1.3% [95% CI: 0.5%-2.5%] (*I*^2^=0%), respectively. The mean difference in the EoE Endoscopic Reference score to placebo was -0.56 [95% CI: -0.91, -0.21].

**Conclusions:**

Over 1/3 of patients in EoE trials clinically respond to placebo and this is associated with trial design factors such as randomization ratio and trial population. Objective endoscopic and histologic measures are associated with lower placebo responses. These findings highlight the need to re-evaluate clinical trial endpoint configurations in EoE

Table 1. Pooled proportion of placebo patients achieving response

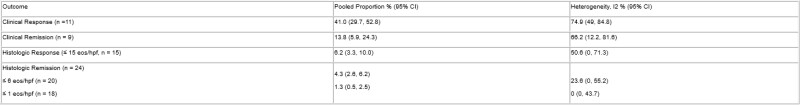

Abbreviations: CI confidence interval; eos eosinophils; hpf high power field

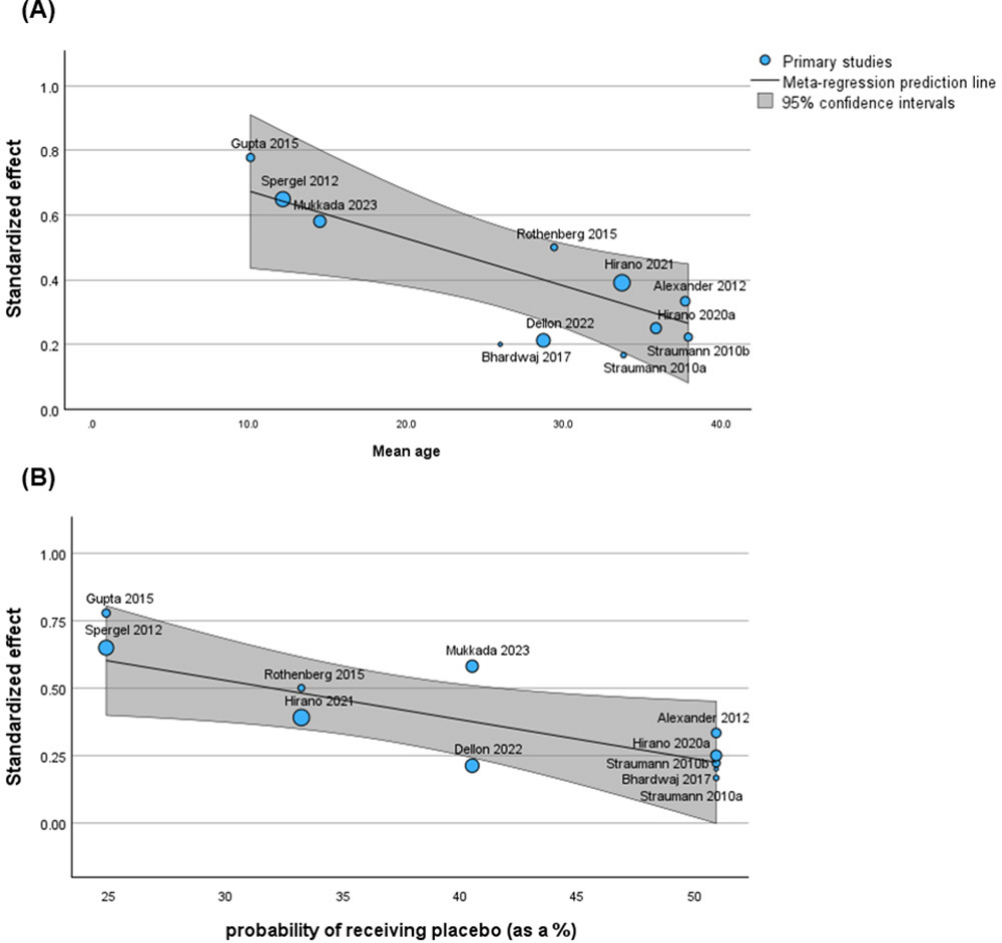

**Figure 3**. Association between mean age of trial participants (A) and probability of receiving placebo (B) with proportion of clinical responders to placebo

**Funding Agencies:**

None

